# Dicer suppresses cytoskeleton remodeling and tumorigenesis of colorectal epithelium by miR-324-5p mediated suppression of HMGXB3 and WASF-2

**DOI:** 10.18632/oncotarget.18218

**Published:** 2017-05-25

**Authors:** Li Na Sun, Cheng Xing, Zheng Zhi, Yao Liu, Liang-Yan Chen, Tong Shen, Qun Zhou, Yu Hong Liu, Wen Juan Gan, Jing-Ru Wang, Yong Xu, Jian Ming Li

**Affiliations:** ^1^ Department of Pathology and Pathophysiology, Soochow University Medical School, Suzhou, People’s Republic of China; ^2^ Department of Pathology, Baoan Hospital, Southern Medical University, Shenzhen, People’s Republic of China; ^3^ Department of Pathophysiology, Nanjing Medical University, Nanjing, People’s Republic of China

**Keywords:** dicer, miRNA, cytoskeleton remodeling, tumorgenesis, colorectal cancer

## Abstract

Emerging evidence indicates that microRNAs, a class of small and well-conserved noncoding RNAs, participate in many physiological and pathological processes. RNase III endonuclease DICER is one of the key enzymes for microRNA biogenesis. Here, we found that DICER was downregulated in tumor samples of colorectal cancer (CRC) patients at both mRNA and protein levels. Importantly, intestinal epithelial cell (IEC)-specific deletion of Dicer mice got more tumors after azoxymethane and dextran sulfate sodium (DSS) administration. Interestingly, IEC-specific deletion of Dicer led to severe chronic inflammation and epithelium layer remodeling in mice with or without DSS administration. Microarray analysis of 3 paired Dicer deletion CRC cell lines showed that miR-324-5p was one of the most significantly decreased miRNAs. In the intestinal epithelium of IEC-specific deletion of Dicer mice, miR-324-5p was also found to be markedly reduced. Mechanistically, miR-324-5p directly bound to the 3′untranslated regions (3′UTRs) of HMG-box containing 3 (HMGXB3) and WAS protein family member 2 (WASF-2), two key proteins participated in cell motility and cytoskeleton remodeling, to suppress their expressions. Intraperitoneal injection of miR-324-5p AgomiR (an agonist of miR-324-5p) curtailed chronic inflammation and cytoskeleton remodeling of colorectal epithelium and restored intestinal barrier function in IEC-specific deletion of Dicer mice induced by DSS. Therefore, our study reveals a key role of a DICER/miR-324-5p/HMGXB3/WASF-2 axis in tumorigenesis of CRC by regulation of cytoskeleton remodeling and maintaining integrity of intestinal barriers.

## INTRODUCTION

RNase III endonuclease DICER, a key player in the biogenesis of microRNAs (miRNAs), has been widely studied in many physiological and pathological programs including cancer. Reduced expression of DICER is associated with poor prognosis in many types of cancers including lung cancer [1] , breast cancer [2] and colorectal cancer (CRC) [3] . In contrast, evidences also suggest an association between DICER down-regulation and poor prognosis in laryngeal squamous cell carcinoma [4] and primary cutaneous T cell lymphomas [5] . Further compounding issue are the findings that either high expression [6] or low expression of DICER [3] can be associated with poor survival of CRC patients. Recently, increased inflammatory cell infiltration and reduction of goblet cells, indicative of a more inflammatory microenvironment in the intestine epithelial layer was found in mice with intestinal epithelial cell (IEC)-specific deletion of Dicer [7] . However, the molecular mechanisms whereby DICER contributes to the pathogenesis of CRC remain insufficiently delineated.

MiRNAs are a class of single-stranded sequences (∼22nt) that can either mediate mRNA degradation or prevent mRNA translation [8] , and have been implicated in the generation and progression of multiple types of cancers [9–11] . As DICER plays an essential role in the biogenesis of miRNAs, the development of in vitro and in vivo Dicer deletion models has paved the way for investigating the key downstream miRNAs involved. For instance, the miRNA Let-7 has been implicated in macrophage activation and anti-tumour immunity [12] . The role of miR-494 in the regulation of breast cancer stemness has also been identified [13] . To date, the miRNAs downstream of DICER that can contribute to CRC pathogenesis remains enigmatic prompting us to look into this matter using a similar strategy.

Persistent chronic inflammation is a general inducer in colorectal tumorigenesis, as patients with Crohn’s disease and ulcerative colitis have an increased risk of suffering from colitis-associated colorectal cancer [14, 15] . Intestinal barrier integrity is key to maintaining intestinal homeostasis and preventing intestinal microbiota and their products (endotoxins, peptidoglycans) and cytokines from entering the systemic circulation [16] . Cytoskeleton remodeling in IECs and disruption of intestinal barrier integrity leads to increased susceptibility not only to infections but also to the development of inflammatory associated colorectal cancer [17] . However, the evidence that links the key miRNAs to cytoskeletal remodeling in IECs and intestinal barrier deregulation is still lacking.

Herein, we found DICER is a tumor suppressor in CRC using clinical samples from CRC patients and an azoxymethane (AOM) plus DSS induced mouse CRC model. Importantly, DICER deletion led to cytoskeleton remodeling and disruption of intestinal barrier by downregulation of miR-324-5p, which targets HMGXB3 and WASF-2. Based on these findings, we propose that DICER/miR-324-5p/ HMGXB3/WASF-2 axis plays a dominant role in cytoskeleton remodeling, intestinal barrier integrity maintaining and CRC tumorigenesis.

## RESULTS

### DICER is a tumour suppressor in colorectal cancer

To understand the clinical significance of DICER in CRC, DICER expression levels were evaluated in clinical samples of CRC patients. In 78% (53/68) of the clinical CRC samples, DICER mRNA levels were reduced in the tumour tissues compared with their counterpart adjacent tissues (Supplementary Figure S1A). Using public data from Skrzypczak et al. [18] , we also found that DICER1 expression was lowest in colon adenocarcima, followed by adenoma and normal colon tissues (Supplementary Figure S1C). Furthermore, western blots showed that protein levels of DICER were significantly down-regulated in the tumour samples compared with normal counterparts (Supplementary Figure S1B). Immunochemistry staining further showed that DICER expression in tumour samples was down-regulated in patients with CRC at more advanced stages (Supplementary Figure S1D and E).

To further define the role of DICER in CRC, we established an AOM and DSS induced CRC mice model using IECs specific Dicer deficiency mice. Unexpectedly, most of the mice with homozygous Dicer deficiency in IECs (Dicer^loxp/loxp^&Villin^Cre^ mice) could not survive beyond one month (Supplementary Figure S2A), so heterozygous mice (Dicer^loxp/+^&Villin^Cre^ mice) were used for the remainder of the experiments. Examination of intestinal gross morphology showed that there were more and larger colorectal tumors developed in the Dicer^loxp/+^&Villin^Cre^ mice than the wild type mice (Supplementary Figure S2B-E). In addition, more advanced lesions were formed in the Dicer^loxp/+^&Villin^Cre^ mice than in the wild-type littermates (Supplementary Figure S2F). Collectively, our study indicates that DICER functions as a suppressor in colorectal tumorigenesis.

### IECs specific Dicer deficiency mice are more fragile to develop severe intestinal epithelial injury and inflammatory response with or without DSS administration

**Figure 1:**
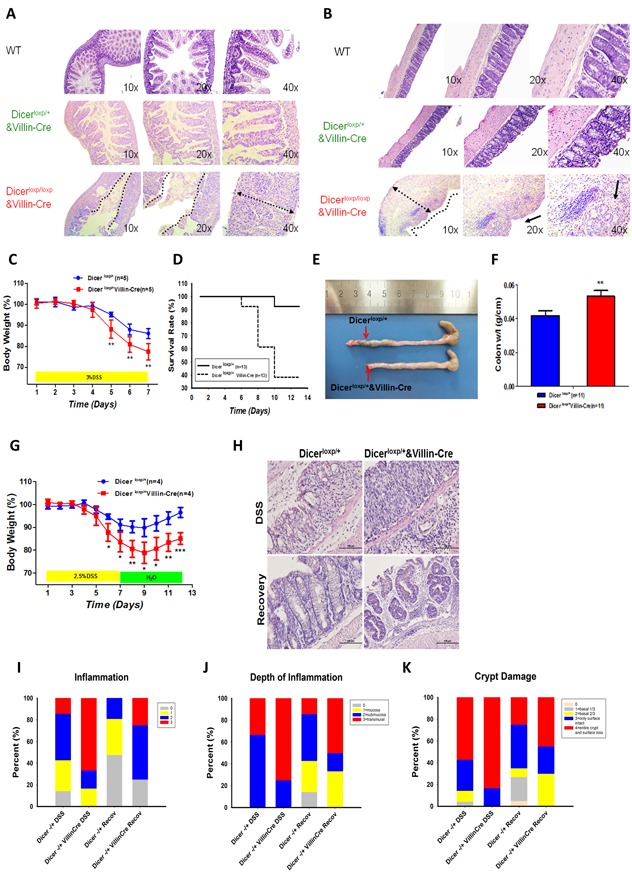
Dicer heterozygous mice are prone to DSS induced colitis **A.**-**B.** H&E staining the small intestine (A) or large intestine (B) of WT mouse (upper panel), Dicer^loxp/+^&Villin^Cre^ mouse (middle panel), and Dicer^loxp/loxp^&Villin^Cre^ mouse (bottom panel) respectively. **C.**-**F.** Dicer^loxp/+^ mice (*n* = 5, blue) and Dicer^loxp/+^&Villin^Cre^ mice (*n* = 5, red) were used in acute colitis model. Body weight (C) results and survival days (D) were recorded; the length of the intestine (E) and the colon weight length radio (F) are presented. Statistical significance was determined using a two-tailed, unpaired Student’s *t-test*; *P* < 0.01. **G.**-**K.** Dicer^loxp/+^ mice (n = 4, blue) and Dicer^loxp/+^&Villin^Cre^ mice (*n* = 4, red) were used in sub-acute colitis mouse models. Mice body weight (G) was recorded. Morphology of the intestine was observed by H&E staining (H) (magnification, × 400). Score of the inflammation degree (I), the depth of inflammation (J) and crypt damage (K) was conducted.

Although few homozygous IECs specific Dicer deficiency mice (Dicer^loxp/loxp^&Villin^Cre^) survived for no more than one month, severe intestinal epithelial injury and inflammatory response in both small intestine (Figure 1A) and large intestine (Figure 1B) were observed in those that survived.

To further investigate the role of Dicer and its downstream miRNAs in gut homeostasis, acute and sub-acute colitis mouse models [19] were produced in Dicer^loxp/+^&Villin^Cre^ mice. In the acute colitis mouse model wherein 6 week-old mice were administered via drinking water with 3% DSS for 7 days, Dicer^loxp/+^&Villin^Cre^ mice began to lose more weight at day 5 compared to their wild-type littermates (Figure 1C). This was accompanied by a remarkable reduction in survival rate (Figure 1D) and in colon size (Figure 1E). Quantitation of the weight length radio showed a significant increase in Dicer^loxp/+^&Villin^Cre^ mice (Figure 1F). In addition, in a sub-acute colitis mouse model wherein six week-old Dicer^loxp/+^ and Dicer^loxp/+^&Villin^Cre^ mice were fed with 2.5% DSS in drinking water for 7 days and then 6 days in normal water, Dicer^loxp/+^&Villin^Cre^ mice showed compromised recovery capacity in body weight (Figure 1G). Haematoxylin and eosin (H&E) staining revealed more severe infiltration by inflammatory cells and reduced recovery ability (Figure 1H). Histopathological scoring showed severe inflammation, depth of inflammation and crypt damage in colons of Dicer^loxp/+^&Villin^Cre^ mice (Figure 1I-1K). Together, our observations suggest the crucial role of DICER in maintaining the homeostasis of intestine.

### DICER deletion leads to cytoskeleton remodeling of intestinal epithelial cells *in vitro* and *in vivo*

Dicer^loxp/+^&Villin^Cre^ mice are more fragile to develop severe intestinal epithelial injury and inflammatory response with or without DSS administration, suggesting a fundamental role of DICER and its downstream miRNAs in maintaining the intestinal mucosal barrier. To confirm whether DICER deficiency will impair the integrity of intestine mucosal, we used Rhodamine tagged Phalloidin to anchor F-actin specifically in our in vivo or in vitro models.

**Figure 2:**
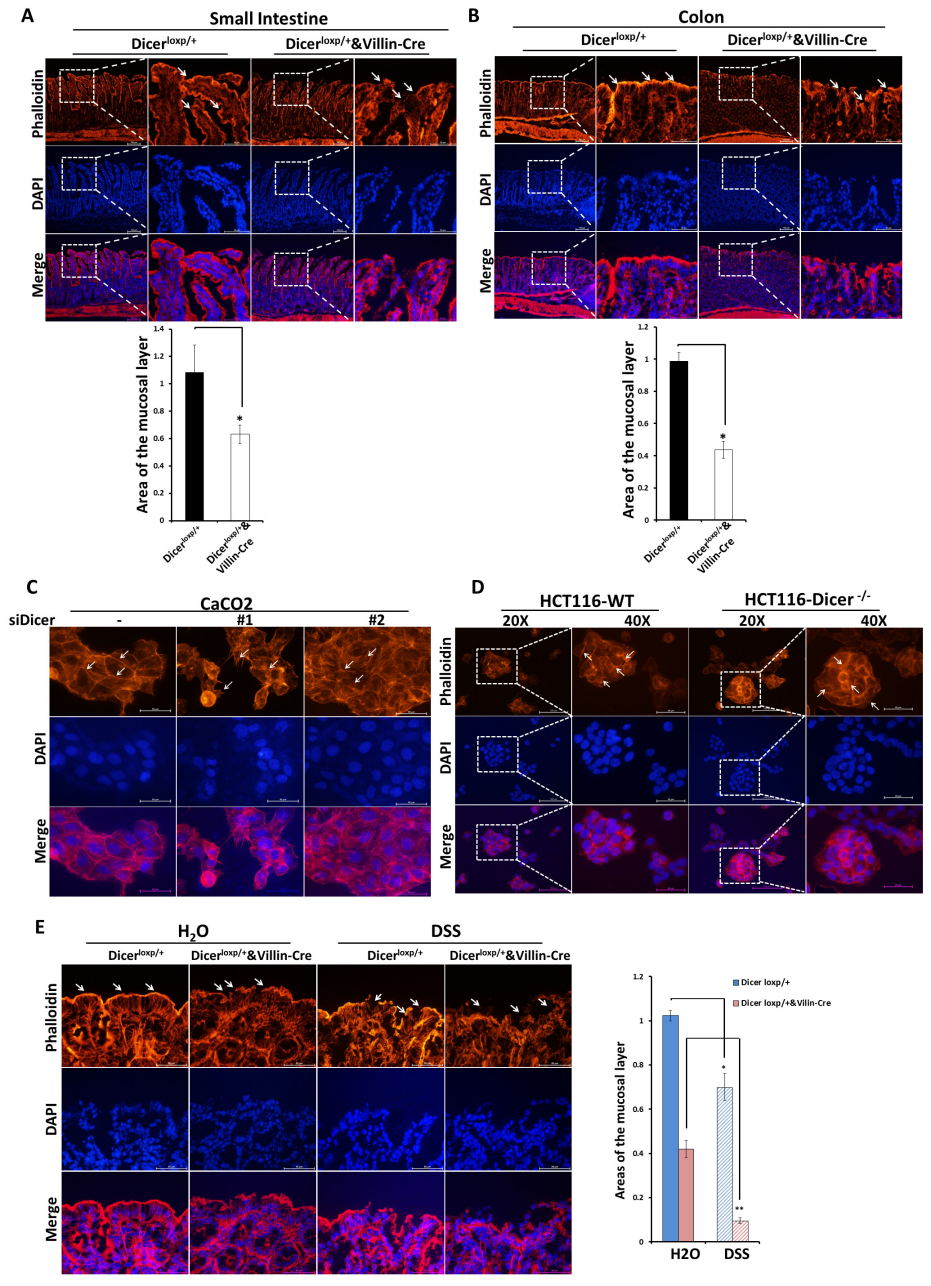
Dicer deletion impairs the stable status of cells *in vivo* and *in vitro* **A.**-**B.** Rhodamine phalloidin staining experiments were performed using frozen sections of small intestine and colon tissues from Dicer^loxp/+^ mice and Dicer^loxp/+^&Villin^Cre^ mice. Representative images for Small intestine (A, Upper panel) and Colon (B, Upper panel) are shown (magnification, left×100, and right × 400). Quantitation of the mucosal area (magnification, × 400) are shown in (A, Bottom panel) and (B, Bottom panel) for small intestine and colon respectively. **C.**-**D.** Caco2 cells transfected with siDICER siRNAs for 48h (C) and HCT116-WT and HCT116-DICER^−/−^cells (D) were stained with Rhodamine phalloidin. Representative images are shown (magnification, × 400). **E.** Dicer^loxp/+^ mice and Dicer^loxp/+^&Villin^Cre^ mice were sacrificed and colon tissues were taken for rhodamine phalloidin staining (left). Representative images are shown (magnification, × 400). Quantitation of the mucosal layer is shown (right). Nuclei were all stained with DAPI (Blue).

Interestingly, even in Dicer^loxp/+^&Villin^Cre^ mice without any stimulation, appreciable cytoskeleton remodeling of intestinal epithelial cells such as partially ribbon fracture, disorganized distribution and down-regulation of F-actin intensity was found both in intestine and colon (Figure 2A and 2B; Upper panel, White Arrow). In contrast, in Dicer^loxp/+^ mice, F-actin was neatly arranged on the edge especially the luminal sides of the enterocytes and the enterocytes tight junctions between cells, creating a barrier to protect the mice from invasion of microorganisms (Figure 2A and 2B; Upper panel, White Arrow). Quantification the area of Phalloidin tagged F-actin showed significantly reduced mucosal barrier area in Dicer^loxp/+^&Villin^Cre^ mice (Figure 2A and 2B, Bottom panel).

Moreover, we also used two in vitro cell systems, Caco2 cells transient transfected with siRNAs targeting DICER (Figure 2C) and HCT116-DICER^−/−^ cell line (Figure 2D), to evaluate the role of DICER in cytoskeleton remodeling. Results showed that DICER downregulation or defiency led to increased F-actin rearrangement, which was characterized by the loss of typical cobble stone appearance and the appearance of rough and irregular cell edge (The positions pointed by white arrows).

Furthermore, in the acute colitis mouse model wherein the mice were fed with 3% DSS in drinking water for 4 days, more fracture at the mucosa layer and less short-column like mucosal cells in Dicer^loxp/+^&Villin^Cre^ mice were found (Figure 2E, white arrow). Quantification assay of mucosa barrier area formed by F-actin showed that the area of the mucosal layer decreased remarkably in Dicer^loxp/+^&Villin^Cre^ mice (Figure 2E), indicating Dicer deletion disrupted intestinal mucosal barrier. The increased cytoskeleton remodeling may cause cells to lose their tight junction and ultimately leads to impaired capacity to maintain the gut homeostasis [20–22] . Collectively, our study demonstrated that DICER deletion led to mucosal barrier disruption and increased cytoskeleton remodeling.

### MiR-324-5p maturation is significantly reduced after DICER deletion

**Figure 3:**
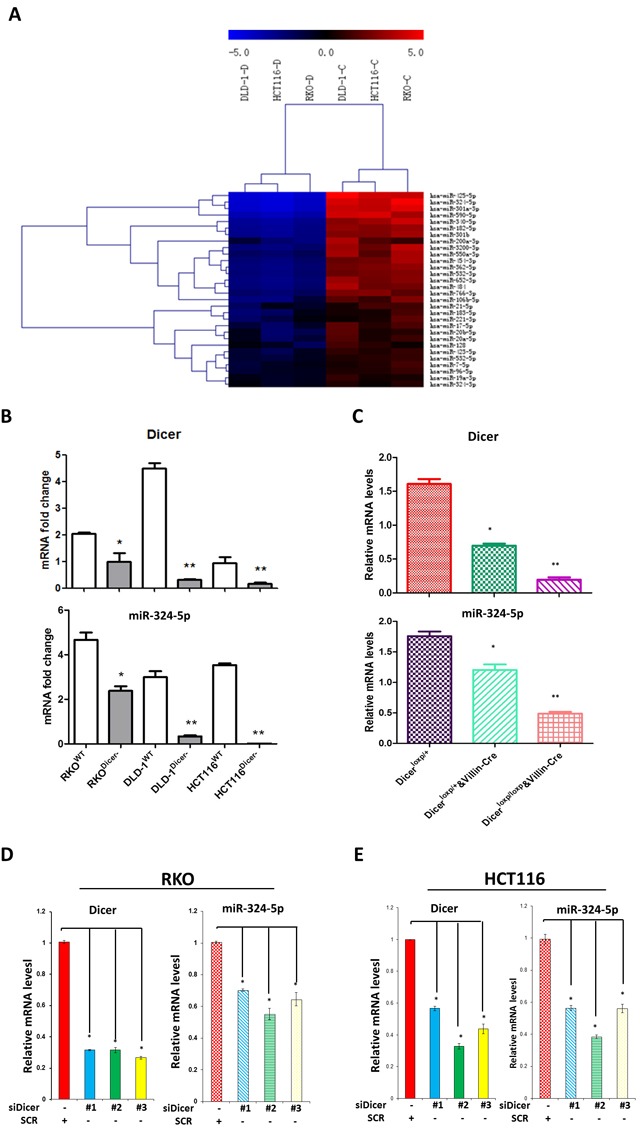
DICER deletion is accompanied with miR-324-5p downregulation **A.** Microarray results of three pairs of DICER-WT and DICER^−/−^ cell lines (Left: DLD, Middle: HCT116, Right: RKO). Downregulated microRNAs are presented. The heat map diagram shows the results of the two-way hierarchical clustering of miRNAs and samples. **B.** Expression of DICER and hsa-miR-324-5p in DICER knockout cell lines. **C.** mRNA levels of Dicer and mmu-miR-324-5p in Dicer^loxp/+^ mice (*n* = 8), Dicer^loxp/+^&Villin^Cre^ mice (*n* = 8) and Dicer^loxp/loxp^&Villin^Cre^ mice (*n* = 8). **D.**-**E.** Cells were transfected with siDICER#1 and #2 for 24h; DICER and has-miR-324-5p expression were analyzed by RT-PCR in RKO (D) and HCT116 (E) cells. * *P* < 0.05. **P < 0.01.

Having established that Dicer deletion led to cytoskeleton remodeling, severe inflammatory response and accelerated tumorigenesis, we asked what downstream miRNAs of Dicer would mainly be responsible for the effects of Dicer deletion on intestinal cytoskeleton remodeling. Three paired DICER-WT and DICER^−/−^ CRC cell lines (RKO, HCT116, and DLD) were exploited to identify the key miRNAs affected by DICER deletion using Human MiRNA Microarray analysis. Expressed data were normalized using the Median normalization (GEO accession number: GSE93177). Finally, hierarchical clustering was performed to determine distinguishable miRNA expression profiles among the samples. Compared with wild-type cells, hsa-miR-324-5p was one of the top two declined microRNAs after DICER deletion and has-miR-324-5p levels were decreased significantly in three DICER deletion cells (Figure 3A, *P* < 0.05). RT-PCR experiments validated decreased has-miR-324-5p expressions in all the three DICER deletion cell lines (Figure 3B). In mice with IECs specific Dicer deficiency (Dicer^loxp/loxp^&Villin^Cre^ and Dicer^loxp/+^&Villin^Cre^), mmu-miR-324-5p mRNA levels were also significantly decreased in the intestine epithelial layer (Figure 3C). Moreover, RT-PCR experiments showed that has-miR-324-5p mRNA levels were also significantly inhibited after siDICER transfection in RKO (Figure 3D) and HCT116 cells (Figure 3E). Together, these data suggest that miR-324-5p is one of the key indispensible DICER downstream microRNAs involved in cytoskeleton remodeling.

### MiR-324-5p is a key player in intestinal injury and inflammatory response after Dicer deletion

**Figure 4:**
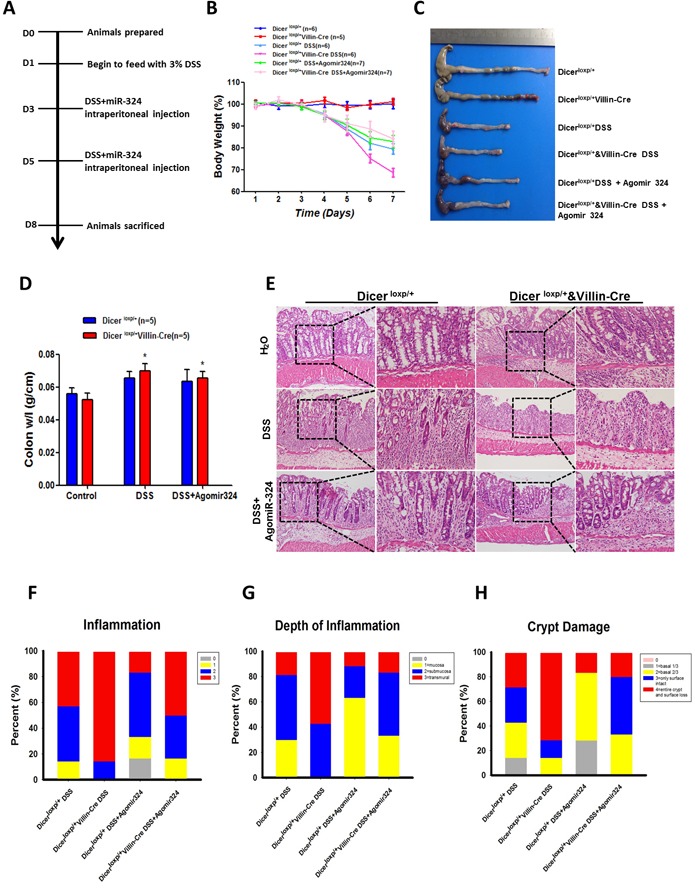
MiR-324-5p agomiR antagonized inflammatory process in mice induced by DSS administration **A.** Mapping of the experiments performed in this figure. **B.** Body weight of the mice was recorded for 7 days. Mice (Dicer^loxp/+^ mice, *n* = 19 and Dicer^loxp/+^&Villin^Cre^ mice, *n* = 18) were divided into 3 groups: Drink water (H_2_O), Drink water containing 3% DSS, 3% DSS water and AgomiR-324-5p intraperitoneal injection. Number of the mice for each group was shown in the figure. **C.** After 7 days, mice were sacrificed and colorectal tissues were pictured. **D.** Quantification of the radio of weight to length was presented (*n* = 5). **E.** HE staining of the colon tissues, representative images are shown (magnification, left×100, and right × 400). **F.**-**H.** Score of the inflammation degree (F), the depth of inflammation (G) and crypt damage (H) was conducted. *P < 0.05.

To explore the functional role of miR-324-5p in maintaining intestinal mucosal barrier, agomiR-324-5p was used to rescue the effects of Dicer deletion on intestinal injury and inflammatory response. In DSS induced colitis mouse model (Figure 4A), intraperitoneal injection of agomiR-324-5p (10nmol) significantly attenuated the weight loss (Figure 4B), shortened size of the colon and rectum (Figure 4C) and decreased radio of weight to length of colon (Figure 4D) induced by 3% DSS in Dicer^loxp/+^&Villin^Cre^ mice but not in Dicer^loxp/+^ mice. HE staining showed agomiR-324-5p alleviated intestinal epithelial injury and the inflammatory cell infiltration, helped repair of the intestinal villus (Figure 4E), which were characterized by the scores for the degree of inflammation (Figure 4F), the depth of inflammation (Figure 4G) and grade of crypt damage (Figure 4H) in Dicer^loxp/+^&Villin^Cre^ mice but not in Dicer^loxp/+^ mice after DSS induction. Taken together, our study demonstrated that miR-324-5p is a key player in maintaining intestinal barriers.

### HMGXB3 and WASF-2 are two direct targets of miR-324-5p

To unravel the mechanisms by which miR-324-5p participates in maintaining intestinal barriers, we employed three miRNA targets predicting websites including TargetScan, miRBase and microRNA.org to find the direct targets of miR-324-5p. HMGXB3 and WASF-2, two proteins involved in cell motility and actin rearrangement were predicted as potential miR-324-5p targets. HMGXB3 belongs to high-mobility group family, which is involved in cell proliferation and migration by promoting cell membrane rearrangement [23] . WASF-2, a member of the WASP/WAVE family, is required for formation of both lamellipodia and filopodia, and dissociation of tight-junction between the cells [24] .

**Figure 5:**
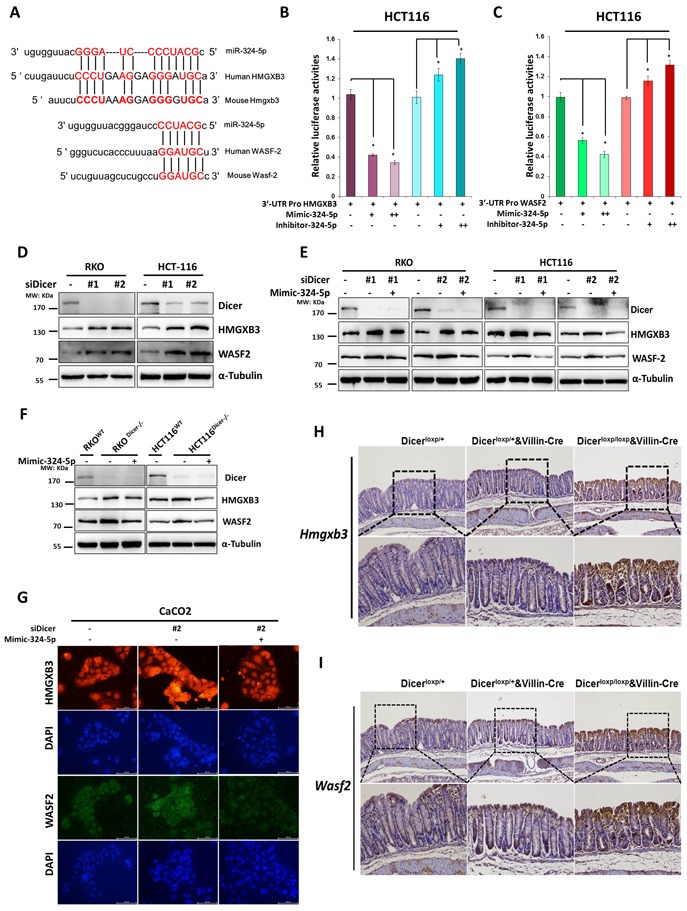
HMGXB3 and WASF-2 are two direct targets for miR-324-5p **A.** Schematic diagram presenting the predicted miR-324-5p binding sequences for HMGXB3 and WASF-2. **B.**-**C.** Luciferase activity of HMGXB3 (B) and WASF-2 (C) 3′UTR constructs were analyzed after being transfected with miR-324-5p mimic or inhibitor. β-gal was transfected as control. **D.** Levels of DICER, HMGXB3 and WASF-2 were examined by using western blot assay after RKO and HCT116 cells transfected with DICER siRNAs. **E.** RKO and HCT116 cells were transfected with DICER siRNAs together with Mimic-324-5p or not, western blotting of DICER, HMGXB3 and WASF-2. **F.**Transfection of Mimic-324-5p in DICER-KO cells to determine the indicated proteins. Western blot experiments all processed 48h after transfection. α-tubulin was chosen as the internal control. **G.** Immunofluorescent staining of HMGXB3 (Red) and WASF-2 (Green) in Caco2 cells transfected with siDICER#2 together with mimic-324-5p or not. Nuclei were stained with DAPI (Blue). Representative images are shown (magnification, ×200). **H.**-**I.** Immunohistochemical staining of H*mgxb3* (H) and W*asf-2* (I) in Dicer^loxp/+^ and Dicer^loxp/+^&Villin^Cre^ mice are presented (magnification, Upper panel × 100; Bottom panel× 200).

As shown in Figure 5A, both the has-miR-324-5p and its mouse homolog (mmu-miR-324-5p) share the identical sequence and are predicted to target WASF-2 and HMGXB3 within the 3′UTR region conservatively (Figure 5A). To further verify whether miR-324-5p could directly bind to the 3′UTR of HMGXB3 and WASF-2, HCT116 cells were co-transfected either miR-324-5p mimic or miR-324-5p inhibitor with 3′UTR promoter reporter plasmids bearing WT target sequences. MiR-324-5p mimic reduced the luciferase activity of both HMGXB3 and WASF-2 reporter plasmids (Figure 5B and 5C). Conversely, the luciferase activities were increased by co-transfected with miR-324-5p inhibitor (Figure 5B and 5C).

To further determine whether down-regulation of DICER could lead to cytoskeleton remodeling by suppression of miR-324-5p and hence restoration of its target proteins, RKO and HCT116 cells were transfected with siRNAs targeting DICER. As shown in Figure 5D, the expressions of HMGXB3 and WASF-2 were significantly increased in RKO and HCT116 cells after DICER was knocked down (Figure 5D). Importantly, the elevated expressions of HMGXB3 and WASF-2 resulted from DICER knockdown could be alleviated by miR-324-5p overexpression (Figure 5E). Similar results could be found in two paired DICER deletion cell lines (RKO and HCT116) (Figure 5F). Immunofluorescence assay showed the similar results (Figure 5G).

In addition, immunohistochemistry staining showed that the increased expressions of *Hmgxb3* and *Wasf-2* was observed in Dicer^loxp/+^&Villin^Cre^ mice or in Dicer^loxp/loxp^&Villin^Cre^ mice compared with Dicer^loxp/+^mice (Figure 5H and 5I). In general, our study demonstrates that HMGXB3 and WASF-2 are two direct targets of miR-324-5p in vitro and in vivo.

### A key role of DICER/MiR-324-5p/HMGXB3/WASF-2 axis in maintaining the intestinal mucosal barrier

**Figure 6:**
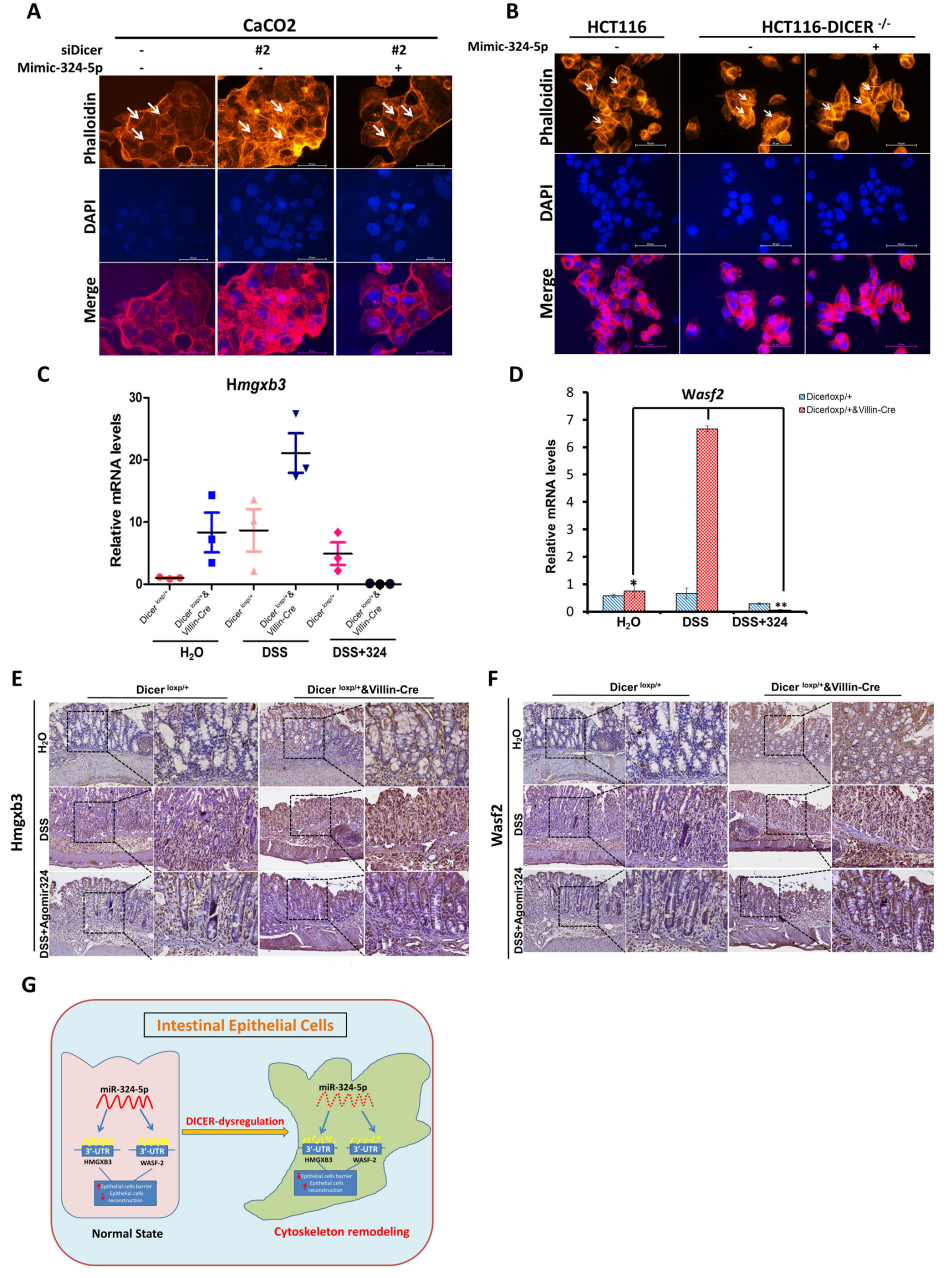
MiR-324-5p protects intestinal mucosal integrity through DCIER/ miR-324-5p/HMGXB3/WASF-2 axis **A.**-**B.** Rhodamine phalloidin staining of Cacao2 cells was performed after 48h transfection with siDICER#2 and together with mimic-324-5p or not. (B) HCT116-WT and HCT116-DICER^−/−^cells were stained by rhodamine phalloidin after transfected with NC/mimic-324-5p. Nuclei were stained with DAPI (Blue). Representative images are shown (magnification, × 400). **C.**-**D.** RT-PCR experiments were performed to measure Hmgxb3 (C) and Wasf-2 (D) mRNA levels. **E.**-**F.** Immunohistochemistry experiments were performed to test Hmgxb3 (E) and Wasf-2 (F) protein levels. **G.** A schematic model displaying the protective role of DICER/miR-324-5p in maintaining the intestinal epithelial integrity by targeting HMGXB3 and WASF-2.

We have found that DICER/miR-324-5p/HMGXB3/WASF-2 axis exists in vitro and in vivo. Next, we asked whether the axis plays the key role in maintaining the intestinal mucosal barrier. We used the rhodamine phalloidin assay to examine the state of F-actin remodeling in colorectal cell lines. Cell polarity was significantly impaired as some of the tight-junction between the cells was diminished after DICER knockdown in Caco2 cells (Figure 6A) or DICER deletion in HCT116 cells (Figure 6B). And, miR-324-5p mimic can effectively rescue the effects of DICER knockdown (Figure 6A) or deletion (Figure 6B) on cell polarity and cell tight-junction. Furthermore, in DSS induced colitis mouse model, AgomiR-324 by intraperitoneal injection significantly rescued the regulatory effects of Dicer on expressions of *Hmgxb3* and *Wasf-2* as evidenced by RT-PCR (Figure 6C and 6D) and immunohistochemistry staining (Figure 6E and 6F). Our results therefore revealed a key role of DICER/MiR-324-5p/HMGXB3/WASF-2 axis in maintaining the intestinal mucosal barrier, suggesting a new strategy for colitis and colitis associated CRC.

## DISCUSSION

DICER, known as the RNase III endonuclease, plays a key role in the biogenesis of microRNAs (miRNAs), has been widely studied in many processes including mammalian embryogenesis [25, 26] , DNA repair [27] , genome stability [28] and different types of cancers. Nevertheless, the role of Dicer in cancer including CRC is still uncertain or even controversial.

Here, we found Dicer as a tumour suppressor in CRC using both clinical samples of CRC patients and in vivo mouse CRC model with IECs specific deletion of Dicer. We found that Dicer was downregulated in tumour samples of CRC patients at both mRNA and protein levels. Importantly, in AOM and DSS induced mouse CRC model, more tumours were developed in mice with IECs specific deletion of Dicer (Dicer^loxp/+^&Villin^Cre^ mice). Although Dicer has been suggested as a tumour suppressor for a long time [29, 30] , the role of Dicer in CRC is still uncertain. Both down-regulation and overexpression of DICER in clinical samples of CRC were reported [31–33] . These paradoxical clinicopathologic studies suggest more functional evidences to know the precision role of Dicer in CRC. In vivo and in vitro experimental models of Dicer deletion are useful tools to uncover the function of Dicer and the miRNA processing pathway in the context of CRC. Using these models, a recent paper found that haploinsufficient specific deletion of *Dicer1* in intestinal epithelial cells promotes tumorigenesis in AOM and DSS induced mouse CRC model [7] , supporting Dicer as tumour suppressor in CRC. However, the key program and miRNAs regulated by Dicer in CRC are still unclear.

Here, we uncover a key role of Dicer in cytoskeleton remodeling of intestinal epithelial cells. Cytoskeleton remodeling of intestinal epithelial cells, accompanied with severe intestinal epithelial injury and inflammatory response, was found in homozygous or heterozygous Dicer deletion mice with or without DSS administration. The cytoskeleton is a crucial component to regulate the intestinal mucosal barrier by controlling the assembly and function of epithelial adherens and tight junctions (AJs and TJs). Therefore, cytoskeleton remodeling of intestinal epithelial cells will be the key step for gut inflammatory response to any stress or inflammation. Interestingly, a recent study showed that Dicer is involved in formation and maintenance of cell-cell junctions of mouse seminiferous epithelium [34] . Our study suggested that intestinal epithelial cells specific ablation of Dicer led to uncontrolled inflammatory response in gut by destroying intestinal barrier integrity and the homeostasis of intestine, and then contributed to tumorigenesis.

More importantly, we found a key mechanism that Dicer suppresses cytoskeleton remodeling of colorectal epithelium by miR-324-5p mediated suppression of HMGXB3 and WASF-2. As one of most abundant miRNAs, miR-324-5p is significantly regulated by Dicer and has a key role in maintaining intestinal epithelial integrity and intestinal homeostasis. We found that HMGXB3 and WASF-2 are two direct targets involved in cytoskeleton remodeling of colorectal epithelium for miR-324-5p. HMGXB3 (HMG-box containing 3), one of the non-canonical high mobility group genes, belonging to the High Mobility Group superfamily, participates in a range of cellular process including cell migration and proliferation [35, 36]. WASF-2, also named WAVE2, has been intensively investigated in promoting cell morphology remodeling by rearrangement of F-actin [37, 38]. Our results show that intestinal epithelial cells with high levels of HMGXB3 and WASF-2 gain dramatic F-actin redistribution, meanwhile, lose the normal cell tight-junctions. Our results, as summarized in Figure 6G, illustrate a key axis that contains DICER/miR-324-5p/ HMGXB3/WASF-2 in the epithelial cell cytoskeleton remodeling and dysregulated DICER/miR-324-5p/ HMGXB3/WASF-2 exacerbated gut inflammatory response and might lead to tumorigenesis of CRC. Furthermore, administration of miR-324-5p agonist could successfully rescue cytoskeleton remodeling and inflammatory response resulted from Dicer dyregulation, suggesting that miR-324-5p is a potential target for prevention and treatment of CRC.

In summary, we found DICER as a tumour suppressor in CRC using clinical samples and an AOM and DSS induced mouse model with Dicer deletion in IECs. Importantly, we suggest a new DICER/miR-324-5p/HMGXB3/WASF-2 axis plays a dominant role in cytoskeleton remodeling, intestinal barrier integrity maintaining and tumorigenesis.

## MATERIALS AND METHODS

### Mice

Villin promoter-driven Cre recombinase transgenic mice (Villin^Cre^ mice) and mice carrying the floxed allele of Dicer (Dicer^loxp/loxp^ mice) were purchased from the Jackson Laboratory. Villin^Cre^ and Dicer^loxp/loxp^ mice were crossed to obtain intestinal-specific Dicer knockout mice (Dicer^loxp/loxp^&Villin^Cre^ mice). The mice used in the present study were maintained in the barrier facilities at the Laboratory Animal Center in Soochow University (China). All the experiments were performed according to the protocols approved by the Committee on Animal Research of Soochow University.

### Cell cultures and chemicals

The HCT116, SW480, RKO, DLD1 and Caco2 human colon cancer cell lines were purchased from the Cell Bank of the Chinese Academy of Sciences (Shanghai, China). HCT116, SW480, RKO and DLD1 were cultured in an RPMI-1640 medium (HyClone) with 10% fetal bovine serum (FBS). Caco-2 were maintained in Dulbecco’s modified Eagle’s medium (DMEM) with 20% FBS. Dicer-knockout cell lines of (HCT116, SW480 and DLD1) were kind gifts of Professor Bert Vogelstein from the Sidney Kimmel Comprehensive Cancer and Howard Hughes Medical Institute. For in vivo studies, the mmu-miR-324-5p agomir (modified miR-324-5p mimic) and the negative control were from RiboBio (Guangzhou, China). MiR-324-5p mimic and inhibitor were purchased from RiboBio (Guangzhou, China). Rhodamine Phalloidin was purchased from Cytoskeleton, Inc.

### Plasmids, transient transfection and luciferase assay

The luciferase reporter gene vectors including HMGXB3 and WASF-2 3′ untranslated region (3′UTR) sequences of the human miR-324-5p binding sites were constructed. Primers (HMGXB3F: GCCAGGCTGTTGTACAGGGA, R: AAGCTTGGGGTGG AGAGGAC; WASF-2 F: ACCCAATGCAGGTAATCCTG, R: CATGTGTATACA GTTACATGG) of HMGXB3 and WASF-2 3′ UTR including miR-324-5p binding sites were amplified respectively. The PCR products were digested and transferred into pMIR-REPORT vector to construct the recombinant pMIR-promoter-HMGXB3 3′UTR-wild type and pMIR- promoter-WASF-2 3′UTR-wild type respectively. siRNAs for human DICER, #1: GGAAGAGGCUGACUAUGAA, and #2: UGCUUGAAGCAGCUCUGGA. Transient transfections were performed with Lipofectamine 2000 (Invitrogen) following the manufacture’s protocol. Cells were lysed 24-48h after transfection and fluorescence intensity was assayed using a luciferase reporter assay system (Promega). Each transfection also included β-gal for normal control. Experiments were performed in triplicate wells and repeated at least three times.

### Protein extraction and western blot

Whole cell protein was extracted using RIPA buffer (150mM NaCl, 50mM Tris pH7.4, 1mM EDTA, 0.1% Triton X-100) supplemented with a protease inhibitor tablet (Roche). Western blot analyses were performed with anti-DICER1 (Santa Cruz), anti-HMGXB3 (Bioss Antibodies, China), anti-WASF2 (Santa Cruz), anti-a-tubulin (Invitrogen) antibodies.

### Phalloidin staining

Prepared frozen section or the transfected cells for the following staining. Slides or cells were then fixed, permeabilized at room temperature and then incubated with 100nM rhodamine phalloidin in the dark for 30 min. Counterstain the nuclei for 30s with DAPI and seal the slides in anti-fade mounting media. Store the slides in the dark at 4°C before observation.

### Statistical analysis

The results are presented as the mean± standard deviation. Statistical significance was determined by Student two-tailed *t* test. *P-*value < 0.05 was considered significant and marked with *. Each experiment was performed in at least three independent experiments. Additional methods employed in this study are described in Supplementary materials and methods (see supplementary material).

## SUPPLEMENTARY MATERIALS FIGURES


