# Supplementary material for: Dicer suppresses cytoskeleton remodeling and tumorigenesis of colorectal epithelium by miR-324-5p mediated suppression of HMGXB3 and WASF-2
Source: Oncotarget. 2017 May 25;8(34):55776–89. doi: 10.18632/oncotarget.18218 (PMC5593523; doi:10.18632/oncotarget.18218)
Supplement: Supplementary file 1 [file oncotarget-08-55776-s001.pdf]

Dicer suppresses cytoskeleton remodeling and tumorigenesis of colorectal epithelium by miR-324-5p mediated suppression of HMGXB3 and WASF-2

Supplementary material

Supplementary materials and methods

RNA extraction and qPCR

Total RNA was extracted using TRIzol Reagent (Takara). Reverse-transcription reactions were performed using a SuperScript First-strand Synthesis System (Invitrogen). Quantitative PCR reactions were performed using SYBR Green PCR mater mix (Applied Biosystems). The mRNA expression of miR-324-5p was determined using Bulge-loop™ miRNA qRT-PCR Primer Sets (one RT primer and a pair of qPCR primers for each set), designed by RiboBio (Guangzhou, China). Real-time PCR reactions were performed on an ABI STEPONE Plus (Life Tech) using the $2^{-\Delta\Delta Ct}$ method.

DSS-induced colitis model

Mice (6-8weeks old, male) were given 2.5% dextran sodium sulphate (DSS) in their drinking water for 7 days, followed by regular water for 6 days to allow them to recover. The mice were weighed daily and were sacrificed after 13 days. Colon tissue and small intestinal tissue were collected for evaluation of inflammation and standard histopathological assessments. To evaluate colitis, paraffin-embedded colon sections were stained with H&E and then inflammation and crypt damage were quantified and given a score (range 0–4), as indicated. Similarly, frozen sections were prepared and stained with phalloidin and then the area of mucosal barrier was quantified.

Inflammation associated-colon tumor model

Mice (6-8 weeks old) were injected intraperitoneally with10 mg/kg azoxymethane (AOM; Sigma, A5486) every other two weeks after their first injection. Similarly, 2.0% dextran sulfate sodium (DSS, MP Biomedicals) was mixed in the drinking water for 7 days, and then followed by 14 days of regular water. This cycle was repeated triplicate and mice were sacrificed after the last cycle. Body weight was checked every2 days. Induced colon tumors were counted and sized. The macroscopic images were taken. Colon tissue and small intestinal tissue were collected for evaluation of inflammation and standard histopathological assessments. Histological inflammatory scoring of the colon tissues was performed in a blinded manner as reported.

Immunofluorescence Microscopy

Cells were fixed with 4% formaldehyde for 10 min and then blocked with 5% bovine serum albumin (BSA). Cells then incubated with indicated primary antibodies overnight. Washed the dishes with phosphate buffered saline (PBS) and incubated the cells with fluorescently-labeled secondary antibodies (Jackson) for 60 min. Finally, added DAIP (Sigma) into the dishes and incubated for 5 min before observation.

Immunohistochemistry

Immunohistochemistry was performed according to a standard protocol. In brief, slides were deparaffinized in xylene and gradually rehydrated in graded ethanol. Antigen retrieval was performed by boiling slides(95~98℃) in citrate buffer(pH 7.0) for 15 min and then cooled to room temperature naturally. To block the endogenous peroxidase, we incubated slides in 10% BSA for 20 min at room temperature. The

slides were incubated subsequently for at least 2h or overnight with primary antibodies and then the secondary antibodies. Color was developed using horseradish peroxidase substrate buffer and DAB^+ chromogen. The slides were slightly counterstained with haematoxylin for 20s, dehydrated gradually in graded ethanol and xylene. Finally, the slides were sealed with a xylol soluble mountant.

Histological Analysis

Formalin-fixed and paraffin-embedded sections were subjected to hematoxylin and eosin staining of 3 μm sections and used standard protocols. Slides were examined by electron microscopy in a blinded fashion. The slides were analyzed separately by two pathologists without knowing the experimental information.

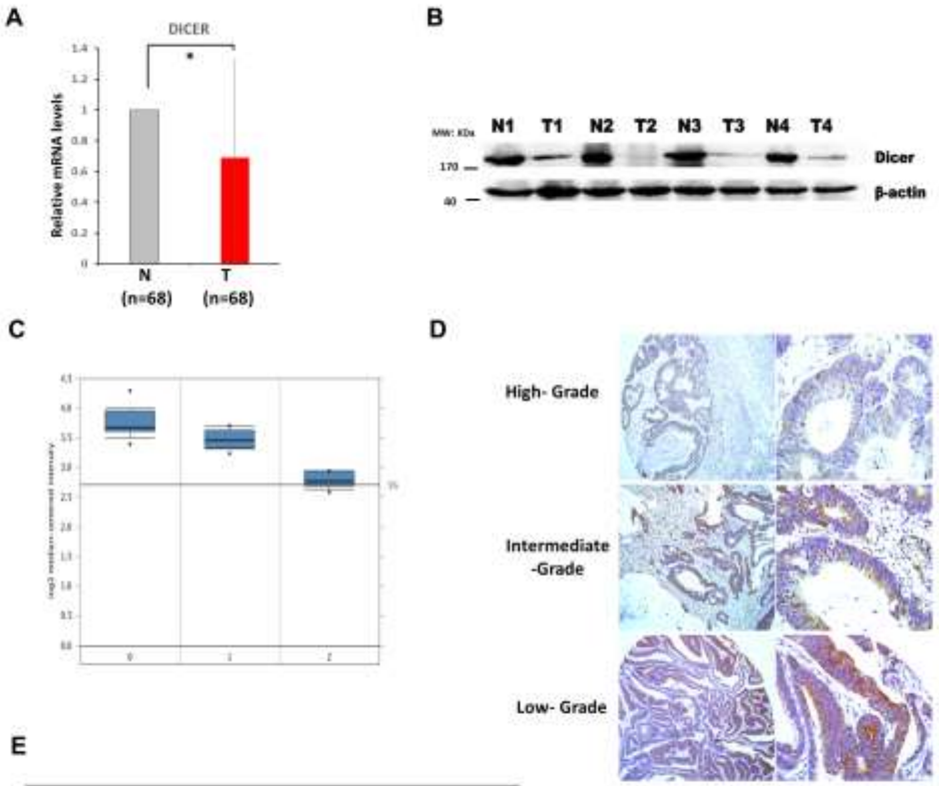

E

Characteristic	Dicer expression		
	Low, n (%)	High, n (%)	*P* value
**Gender**	49	30	0.793
Male	24 (30.38%)	17(21.52%)	
Female	25(31.64%)	13(16.46%)	
**Age(years)**	49	30	0.200
<60	9(11.39%)	4(5.06%)	
>=60	40(50.64%)	26(32.91%)	
**Site**	49	30	0.773
Above rectosigmoid	28(35.44%)	21(26.58%)	
Rectosigmoid and below	21(26.58%)	9 (11.4%)	
**Differentiation**	47	28	0.041
Good	4(5.33%)	6(8%)	
Moderate	21(28%)	18(24%)	
Poor	22(29.34%)	4(5.33%)	
**Depth**	48	29	0.49
Submucosa	1(1.3%)	2(2.6%)	
Muscle	2(2.6%)	2(2.6%)	
Serosa	45(58.44%)	25(32.46%)	
**Lymph status**	45	24	0.001
Negative	24(34.78%)	18(26.09%)	
Positive	21(30.43%)	6(8.7%)	
**Distant metastasis**	49	30	0.389
Yes	2(2.53 %)	0(0%)	
No	47(59.5%)	30(37.97%)	
**TNM stage**			0.227
Stage Ⅰ	3(3.84%)	4(5.14%)	
Stage Ⅱ	26(33.33%)	19(24.36%)	
Stage Ⅲ	18(23.07%)	6(7.7%)	
Stage Ⅳ	2(2.56%)	0(0%)	
**Dukes classification**			0.047
**Dukes A**	0(0%)	3(3.84%)	
**Dukes B**	29(37.18%)	20(25.65)	
**Dukes C**	18(23.07%)	6(7.7%)	
**Dukes D**	2(2.56%)	0(0%)	

Supplementary FigureS1: DICER is a tumour suppressor in colorectal cancer.

(A) DICER mRNA expression in the tumour tissues (Red) and its normal counterparts (Gray) was detected using RT-PCR experiments (N=68). Statistics analysis of DICER mRNA expression. Data are expressed as the mean±SEM, *P*<0.001. (B) DICER

protein expression was analyzed in 4 randomly selected pairs of tissue samples from CRC patients using western blot. N (Normal), T (Tumor). (C) In Skrzypczak et al. study, the expression level of DICER1 in normal colon tissues was the highest (n=10, left), and the expression level of DICER1 in colon adenocarcinoma (n=10, right) was significantly lower than that in colon adenoma (n=10, middle). (D) Immunohistochemistry assays were performed staining DICER using various differentiated grades colorectal cancer tissues. (E) Relationship between expression levels of DICER and various clinicopathologic characteristics (n=68).

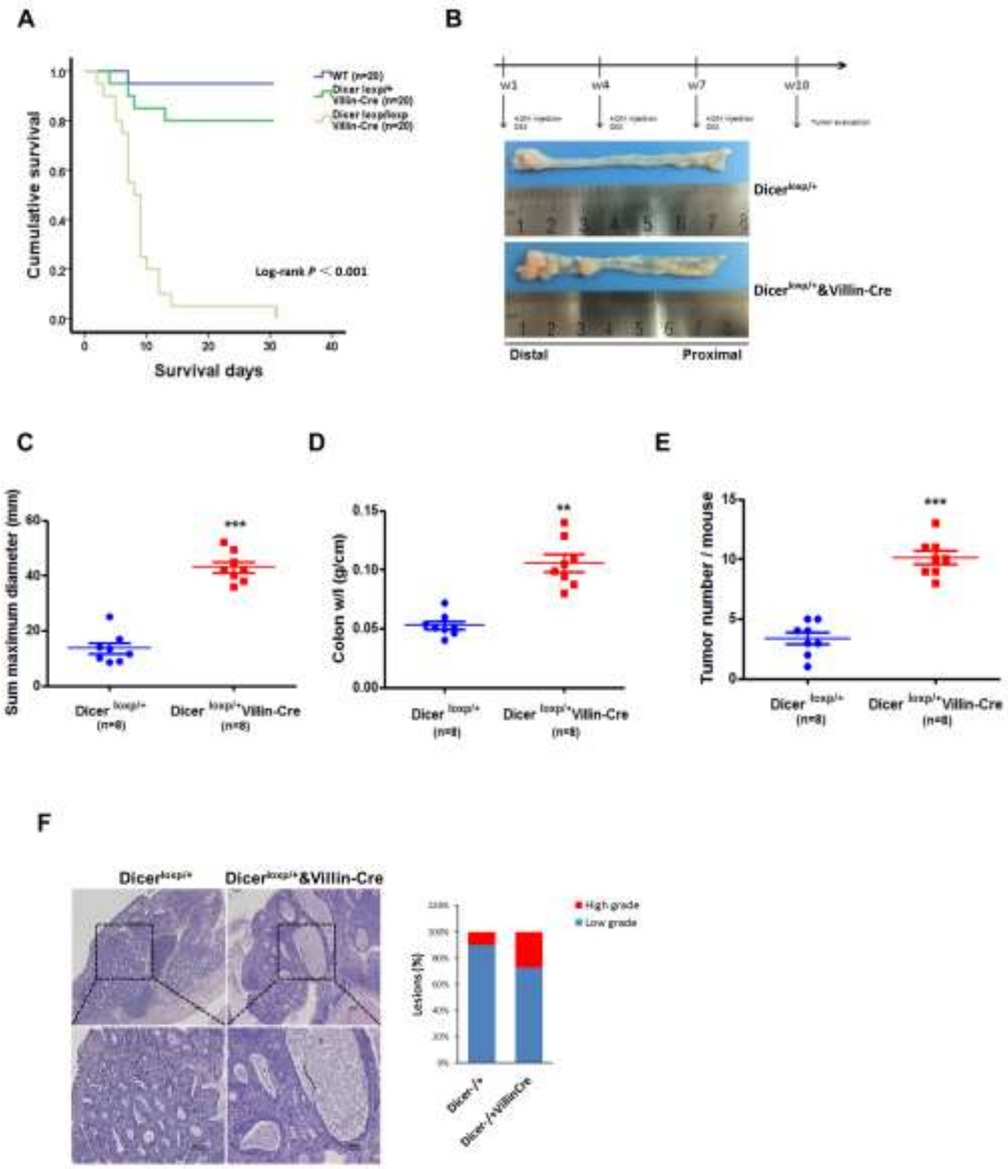

Supplementary FigureS2: Dicer heterozygous mice are more fragile to DSS induced colorectal cancer.

(A) Survival time was record in WT, Dicer $^{loxp/+}$&Villin^{Cre}, and Dicer$^{loxp/loxp}$&Villin^{Cre} mice (n=20). Long-rank test was performed, P<0.001. (B) Inflammation induced colorectal cancer tumorigenesis model was performed using WT (Dicer$^{loxp/+}$) (n=8)

and Dicer$^{loxp/+}$&Villin^{Cre} mice (n=8). Colorectal tissues were presented in the pictures. (C-E) Analysis results of the DSS and AOM induced colorectal tumorigenesis model (n=8). Statistical significance was determined using a two-tailed, unpaired Student's *t-test*; C) Sum of the maximum diameter; $P < 0.001$; D) Colon weight length radio; $^{**}P < 0.001$; E) Tumor number per mouse; $^{***}P < 0.001$. (F) HE staining of the samples from (B) (left), and tumor score results was presented (Right).
